# Anxiety and depression among patient’s companions during admission to the ICU in the Omicron wave of COVID-19: A cross-sectional study in Aleppo University Hospital

**DOI:** 10.1371/journal.pone.0273900

**Published:** 2022-10-31

**Authors:** Sarya Swed, Fateh Kashkash, Sheikh Shoib, Nour Shaheen, Mohamad Nour Nasif, Karam R. Motawea, Ahmed Sallam ElHawary, Yossef Hassan AbdelQadir, Muhammad Mainuddin Patwary, Hidar Alibrahim, Bisher Sawaf, Lina Taha Khairy, Agyad Bakkour, Ali Hadi Hussein Muwaili, Dhuha Hadi Hussein Muwaili, Fatima Abubaker Abdalla Abdelmajid, Nashaat Kamal Hamdy Elkalagi, Mohamed Elsayed, Eman Mohammed sharif Ahmed, Abdullah Khouri, Data Collection group

**Affiliations:** 1 Faculty of Medicine, Aleppo University, Aleppo, Syria; 2 Department of Pulmonology, Aleppo University Hospital, Aleppo, Syria; 3 Department of Psychiatry, Jawahar Lal Nehru Memorial Hospital, Srinagar, Kashmir, India; 4 Faculty of Medicine, Alexandria University, Alexandria, Egypt; 5 Environment and Sustainability Research Initiative, Khulna, Bangladesh; 6 Environmental Science Discipline, Life Science School, Khulna University, Khulna, Bangladesh; 7 Department of Internal Medicine, Syrian Private University, Damascus, Syria; 8 The National Ribat University, Al-Ribat, Sudan; 9 Faculty of Medicine, Albaath University, Homs, Syria; 10 Ivano-Frankivsk National Medical University, Ivano-Frankivsk, Ukraine; 11 University of Medical Sciences and Technology, Khartoum, Sudan; 12 Lecturer in Internal Medicine and Tropical Medicine at Faculty of Medicine Al Arish University, Al Arish, Egypt; 13 Department of Psychiatry and Psychotherapy III, University of Ulm, Ulm, Germany; 14 Nile Valley University, Atbra, Sudan; 15 The President of Department of Pulmonology, Aleppo University Hospital, Aleppo, Syria; King Saud University, SAUDI ARABIA

## Abstract

**Background:**

After the COVID-19 pandemic, anxiety and depression have reached high levels, especially after the last wave, Omicron. Healthcare workers in contact with COVID-19 patients or those who come in contact with them may exhibit high levels of anxiety and depression. Therefore, we aimed to assess anxiety and depression symptoms among ICU companions of COVID-19 patients.

**Methods:**

From 30 November 2021 to 1 March 2022, sixth-year medical students at Aleppo University Hospital conducted interviews with the companions of COVID-19 patients who they brought their patient to the ICU centre as part of a cross-sectional quantitative study using the PHQ-9 and GAD-7 tools to gauge their level of anxiety and depression among companions of COVID-19 patients. The data were analyzed using the SPSS program. In addition, logistic regression models were used to study possible factors of anxiety and depression symptoms during COVID-19.

**Results:**

The total number was 997 participants in contact with COVID-19 patients. The mean score of the depression assessment tool (PHQ-9) in our questionnaire was 9.5 with a range of 0 to 27. At the same time, the anxiety assessment tool (GAD-7) had a mean score of 9.1, ranging from 0 to 21. A binary logistic regression was used to predict the relationship between depression and anxiety and various factors. We found that the companions with medical specialties were substantially less likely to develop anxiety than other companions [AOR = 0.459; 95%CI (0.23–0.9)], in addition females were substantially higher likely to develop depression than males [AOR = 1.322; 95%CI (0.992–1.762)]. 45.4% of companions had moderate to severe anxiety, in additon 50.8% of companions had moderate to severe depression.

**Conclusion:**

Our research reveals that moderate to severe anxiety and sadness are present in roughly half of the COVID19 patients’ companions. Females, people with children, and hard workers were more inclined to feel anxious than others, and those who are not in the medical field were more likely to suffer from depression than others, thus it is critical to assist these groups during the present outbreaks (Omicron and Monkeybox).

## Introduction

COVID-19, also known as Coronavirus disease (COVID), was first identified in December 2019 in Wuhan, China, as a disease caused by the severe acute respiratory syndrome coronavirus (SARS-CoV-2) [1]. Even though COVID-19 is a new strain of coronavirus, it has been associated with illnesses ranging from the common cold to more severe ailments like severe acute respiratory syndrome (SARS) and Middle East respiratory syndrome (MERS) [1]. The Coronavirus infection causes symptoms such as fever, cough, chills, sore throat, nausea, myalgia, diarrhoea, and vomiting [2]. The risk of infection is higher for men with underlying diseases and may result in worse outcomes [2].

COVID-19 pandemic and the public health measures that followed it have been linked to higher rates of mental illness in general [3]. The virus outbreak led to various psychological effects on individuals, communities, nations, and even the entire world [4, 5]. There may be a higher prevalence of the fear of death or illness, feeling helpless at an individual level than at a social level [6]. People’s daily lives have been altered due to the COVID-19 pandemic, resulting in adverse mental health conditions like depression, anxiety, and stress(Depression is a mental condition characterised by chronic sorrow and lack of interest, Anxiety is a sense of discomfort that may range from minor to severe and includes concern or dread, and Stress is a typical bodily response to change that causes physical, emotional, and cognitive reactions.) [7]. Depending on the setting of the outbreak and the study population, COVID-19 infection is associated with varying mental health problems in the general population [8].

Coronavirus Disease 2019 (COVID-19) has profoundly altered social and working environments [9]. Policies of social distance, lockdowns, isolation periods, and anxiety about getting sick, along with suspensions of productive activity, income loss, and fear of the future, negatively impact the mental health of citizens and workers [10].

Mental health of people facing this pandemic scenario can be moderated or worsened by workplace factors [11]. Different occupations have been affected differently by the COVID-19 pandemic. As a result of close contact with infected patients, people accompanying Covid-19 patients during admission are more likely to become infected. Their psychological well-being and performance can be adversely affected by following COVID-19 protocols. Witnessing the suffering and death of patients often has a negative impact on this group’s mental health [12]. Anxiety and/or fear associated with COVID-19 may also negatively affect healthcare job performance and job satisfaction, resulting in frequent absences from work and loss of income [13].

Identifying individuals in the early stages of psychological disorders such as fear, anxiety, depression, or insecurity improves the effectiveness of intervention strategies [14]. Hence, it is essential to establish purposeful mental health interventions early in Syria to address protective factors and the process of successful adaptation to adverse conditions.

Individuals in direct contact with or accompanying patients with COVID-19 infection are at a greater risk of developing psychological disorders, so in this study, we want to assess the levels of anxiety and depression in Covid-19 patients’ companions during the omicron wave at Aleppo University Hospital.

## Methods

### 2.1. Study design

This study is a cross-sectional quantitative study based on an interview approach among companions of COVID-19 patients admitted to the ICU center at Aleppo university hospital from 30 November 2021 to 1 March 2022.

### 2.2. Objectives

This study aims to assess anxiety and depression in COVID-19 patients’ companions during admission to the ICU.

### 2.3. Settings and participants

The target population was those who come with COVID-19 patients to the ICU center at Aleppo university hospital. Inclusion criteria included Syrian nationality, 18 years or older, willing to participate, and able to use the Internet. The exclusion criteria were under 18 years of age, major psychological disorders, and unwillingness to participate in the study.

### 2.4. Informed consent

Prior to the collection of data, informed consent was obtained electronically. We limited the age range for items such as age to individuals over 18 years of age, and we encouraged the respondents to carefully read the instructions before completing the survey. Overall, 997 participants completed the questionnaires, with the data analysis including all of them.

### 2.5. Measurements

#### 2.5.1. Demographic information

The questionnaire consists of questions about age, gender, job, education level, marital status, and an additional question about chronic diseases.

#### 2.5.2. The Patient Health Questionnaire (PHQ-9)

A self-administered version of PRIME-MD, (PHQ)-9 is a depression diagnostic tool based on the DSM-IV depression criteria, a 9-item, self-reported scale that ranges from 0 (not at all) to 3 (very) that asks people whether they have had depressive symptoms in the preceding two weeks (nearly every day). The overall PHQ-9 scores are divided into four categories: 0–4 = "Minimal depression," 0–27 = "Most severe depressive symptoms," and more. "Mild depression," 5–9 "Moderate depression," 10–14. 20–27 = "Severe depression," 15–19 = "Moderately severe depression" [15, 16].

#### 2.5.3. Generalized Anxiety Disorder-7 (GAD-7)

Anxiety Disorder Scale (GAD 7-Scale), which includes a self-reported 7-item questionnaire that asks individuals how often they have been disturbed by the signs in the last two weeks on a 4-point Likert scale ranging from 0 (not at all) to 3. (nearly every day). The GAD-7 total score for the seven questions varies from 0 to 21, with the categories of normal (0–4), mild (5–9), moderate (10–14), and severe (15–21) being used to describe the results [17].

### 2.6. Ethical approval

The study was carried out following the Declaration of Helsinki ethical standards and was approved by the Ethics Committee of Aleppo University Hospital. All participants provided electronic consent.

### 2.7. Data quality assurance and control

Utilizing paper-based questionnaires, data was gathered from the ICU division of the Aleppo University Hospital. The Cronbach alpha value for anxiety and depression, respectively, was determined to be 0.787 and 0.763 after a questionnaire’s reliability and validity were examined. The flow, readability, and clarity of the questionnaire were also evaluated using a pretest, which was conducted on 20 participants but was excluded from the final data collection. Eleven data collectors (sixth-year medical students) and one supervisor (the president of the department of neurology at Aleppo University Hospital) were chosen for data collecting. The supervisor has instructed the data gatherers on how and what information they should gather from the selected data sources in order to maintain data quality. The supervisor verified each day that the data obtained were accurate and consistent. The filled-out information forms were quickly cross-checked with the source data if there seemed to be incompleteness or ambiguity in the recording.

### 2.8. Statistical analysis

Analyses were conducted using SPSS version 20. To demonstrate the percentages and frequencies of research variables, descriptive statistics and inferential statistics (chi-square tests) were used.

To determine the relationship between various factors, bivariate and multivariate analyses were also performed. For multivariable logistic regression, the variables that showed a statistically significant value at a p-value of 0.25 in the bivariate analysis were chosen. The Hosmer and Lemeshow test was used to determine how well the final model fit, and the results for anxiety and depression were 0.3. Multi-collinearity was also evaluated. The degree of association between variables was determined using an adjusted odds ratio with a 95% confidence interval. Statistical significance was determined by a P-value less than 0.05.

## Results

### 3.1. Demographic baseline characteristics

Nine hundred ninety-seven companions of ICU filled our traditional paper survey of admitted COVID-19 patients. The questionnaire was filled almost equally by both sexes, with a slight predominance for males over females (505[50.7%], 492[49.3%], respectively). Most of our sample were smokers (n = 585, 58.7%). 602 (60.4%) of the companions were of moderate economic level. At the same time, high and low economic levels represented 39.6% of the sample (9.7% (n = 97) for high economic levels and 29.9% (n = 298) for low economic levels). Non-medical companions were more than the medical ones by far (959, 96.2% were non-medicals, while only 38 companions [3.8%] were medical personnel). Most of the participants had moderate occupational status followed by hard then easy occupational status (550 [55.2%], 288 [28.9%], and 159 [15.9%], respectively (Table 1).

**Table 1:** Baseline characteristics of the participants in the study sample (N = 997).

Variables Statement.	Number/(Mean+/-SD)	Frequency
Age	39+/-13.5(18–87)	-
Gender		
Female	492	49.3%
Male	505	50.7%
Smoking		
Yes	412	41.3%
No	585	58.7%
If yes, number of smoke cigarettes per day	20.6+/-10.34(1–60)	-
Economic level		
High	97	9.7%
Moderate	602	60.4%
Low	298	29.9%
Maternal status		
Single	320	32.1%
Married	629	63.1%
Widower	21	2.1%
Divorced	27	2.7%
Specialty		
Medical	38	3.8%
Non-medical	959	96.2%
Occupational status		
Easy	159	15.9%
Hard	288	28.9%
Moderate	550	55.2%
Chronic diseases		
Yes	299	30%
No	298	70%
Do you have children?		
Yes	607	60.9%
No	390	39.1%
Who lives with you?		
With family	931	93.4%
Alone	66	6.6%

### 3.2. Assessment of PHQ-9 & GAD-7

The resulting mean score of the depression assessment tool in our questionnaire (PHQ-9) was 9.5 (0.17, 95% CI: 9.15–9.87), ranging from 0 to 27. While the mean score of the anxiety assessment tool (GAD-7) was 9.1 (0.16, 95% CI: 8.83–9.46), ranging from 0 to 21 (Table 2).

**Table 2:** The summary of results (PHQ-9 & GAD-7).

Variables	Mean(SD, 95% CI: Lower-Upper)	Range(Max-Min)
PHQ-9	9.5(0.17, 95% CI: 9.15–9.87)	0–27
GAD-7	9.1(0.16, 95% CI: 8.83–9.46)	0–21

### 3.3. Prevalence of anxiety and predicted factors for anxiety (binary logistic regression) among the study sample

**Fig 1:**
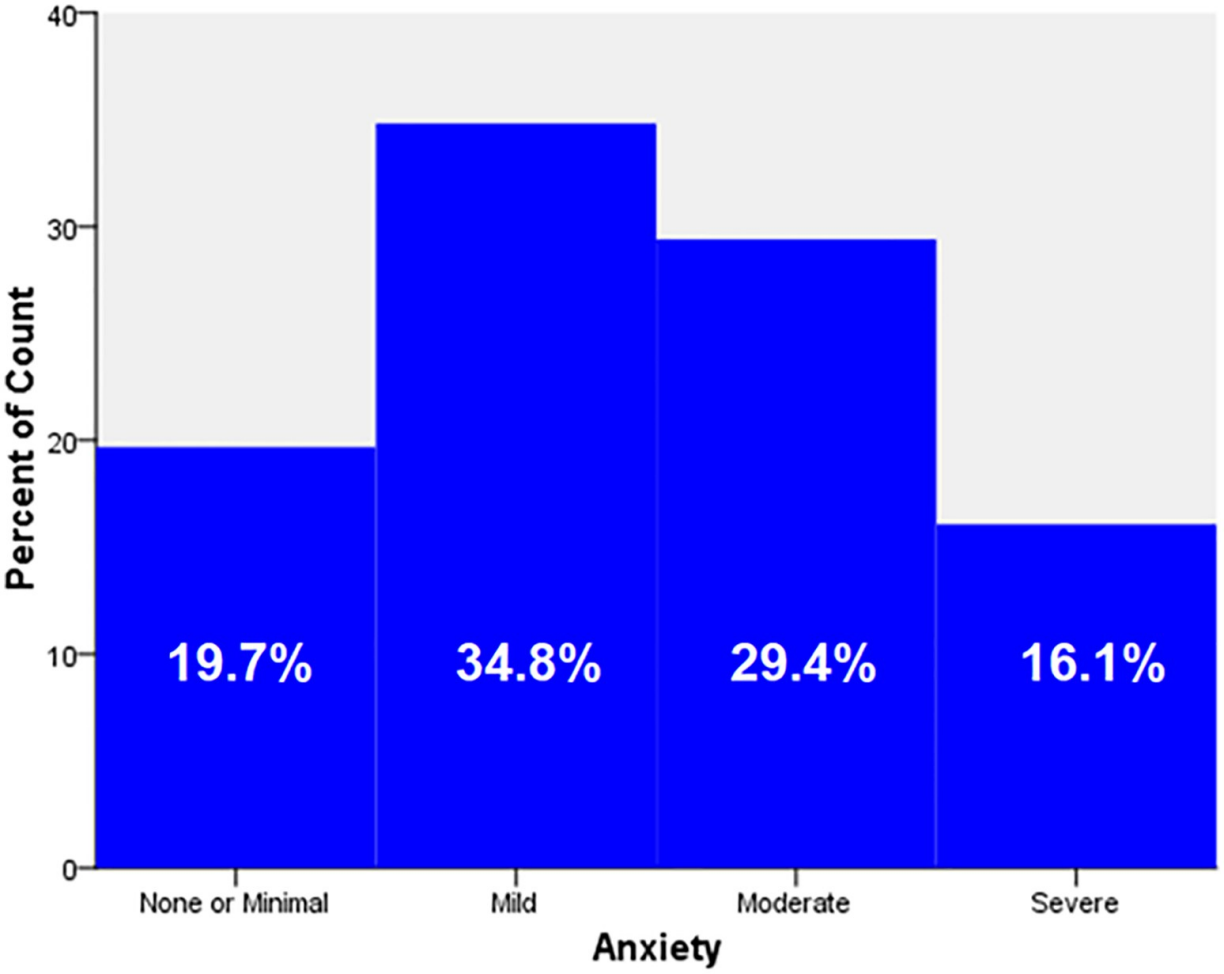
Prevalence of anxiety among the participants in the study sample.

Table 3 displays the respondent’s answers to the GAD-7’s seven items. The following was honestly reported as occurring for a few days, more than half the days, or almost every day by these respondents. excessive anxiety regarding several issues 81.2%); difficulty unwinding (68.8%); fear that something terrible might occur (66.9%); difficulty remaining still due to restlessness (63.3%); sensitivity to irritation or annoyance (76.2%); inability to stop or control worrying (73%); and feeling tense, anxious, or on edge (79.9%). According to our data, 19.7% of companions experienced minimum anxiety, 34.8% had mild anxiety, 29.4% had moderate anxiety, and 16.1% had severe anxiety (Fig 1). To demonstrate the correlation and quantify the relative impact of each independent variable on the total level of anxiety among the companions, a binary logistic regression analysis was conducted. The logistic regression model was not statistically significant, with a p-value of 0.053 for X2(8) = 15.35. Tests by Hosmer and Lemeshow: 8.3 (P = 0.307). The Nagelkerke R Square of the model’s explanation of anxiety-related components was 0.021. The only characteristics that were significantly linked with anxiety among the companions (P-value 0.05) were the specialty and the existence of chronic illness. Companions with medical specialties were substantially less likely to develop anxiety than other companions [AOR = 0.459; 95%CI (0.23–0.9)], and Companions with chronic illness were significantly more likely to acquire anxiety than other companions [COR = 1.371; 95%CI (1.057–1.780)], Table 4.

**Table 3:** Prevalence of anxiety among the participants in the study sample.

		Frequency	Percentage %
Feeling nervous, anxious, or on edge.	Not at all sure	200	20.1%
	Several days	335	33.6%
	Over half the days	219	22.0%
	Nearly every day	242	24.3%
Not being able to stop or control worrying	Not at all sure	269	27.0%
	Several days	324	32.5%
	Over half the days	241	24.2%
	Nearly every day	162	16.3%
Worrying too much about different things.	Not at all sure	259	26.1%
	Several days	307	30.9%
	Over half the days	241	24.2%
	Nearly every day	187	18.8%
Trouble relaxing	Not at all sure	311	31.2%
	Several days	331	33.2%
	Over half the days	193	19.4%
	Nearly every day	161	16.2%
Being so restless that it’s hard to sit still.	Not at all sure	363	36.7%
	Several days	331	33.4%
	Over half the days	183	18.5%
	Nearly every day	113	11.4%
Becoming easily annoyed or irritable	Not at all sure	237	23.8%
	Several days	287	28.8%
	Over half the days	236	23.7%
	Nearly every day	236	23.7%
Feeling afraid as if something awful might happen	Not at all sure	330	33.1%
	Several days	274	27.5%
	Over half the days	201	20.2%
	Nearly every day	190	19.1%
	Not at all sure	1	0.1%

**Table 4:** Factors associated with anxiety among the participants in the study sample.

		Anxiety											
		No Anxiety		Anxiety									
		Frequency	Percentage%	Frequency	Percentage%	P-value	COR	Lower	Upper	P-value	AOR	Lower	Upper
Age	13–17	2	0.2%	5	0.5%	.121				.542			
	18–25	90	9.0%	102	10.2%	.351	.453	.379	2.394	.379	.473	.089	2.504
	26–30	47	4.7%	81	8.1%	.664	.689	.624	3.694	.624	.656	.122	3.538
	31–40	82	8.2%	146	14.7%	.689	.712	.602	3.753	.602	.640	.120	3.418
	41<	162	16.3%	279	28.0%	.658	.689	.529	3.591	.529	.583	.109	3.120
Economic level	Low	109	10.9%	189	19.0%	.551				.786			
	Moderate	233	23.4%	369	37.0%	.536	.913	.992	1.217	.992	.998	.744	1.339
	High	41	4.1%	55	5.5%	.283	.774	.526	1.235	.526	.857	.532	1.380
Specialty	Non-Medical	361	36.2%	598	60.0%								
	Medical	22	2.2%	15	1.5%	.009	.412	.211	.804	.025	.459	.232	.905
Chronic diseases	No	167	16.8%	221	22.2%								
	Yes	216	21.7%	392	39.4%	.018	1.371	1.057	1.780	.166	1.262	.908	1.752

### 3.4. Prevalence of depression and predicted factors for depression (binary logistic regression) among the study sample

**Fig 2:**
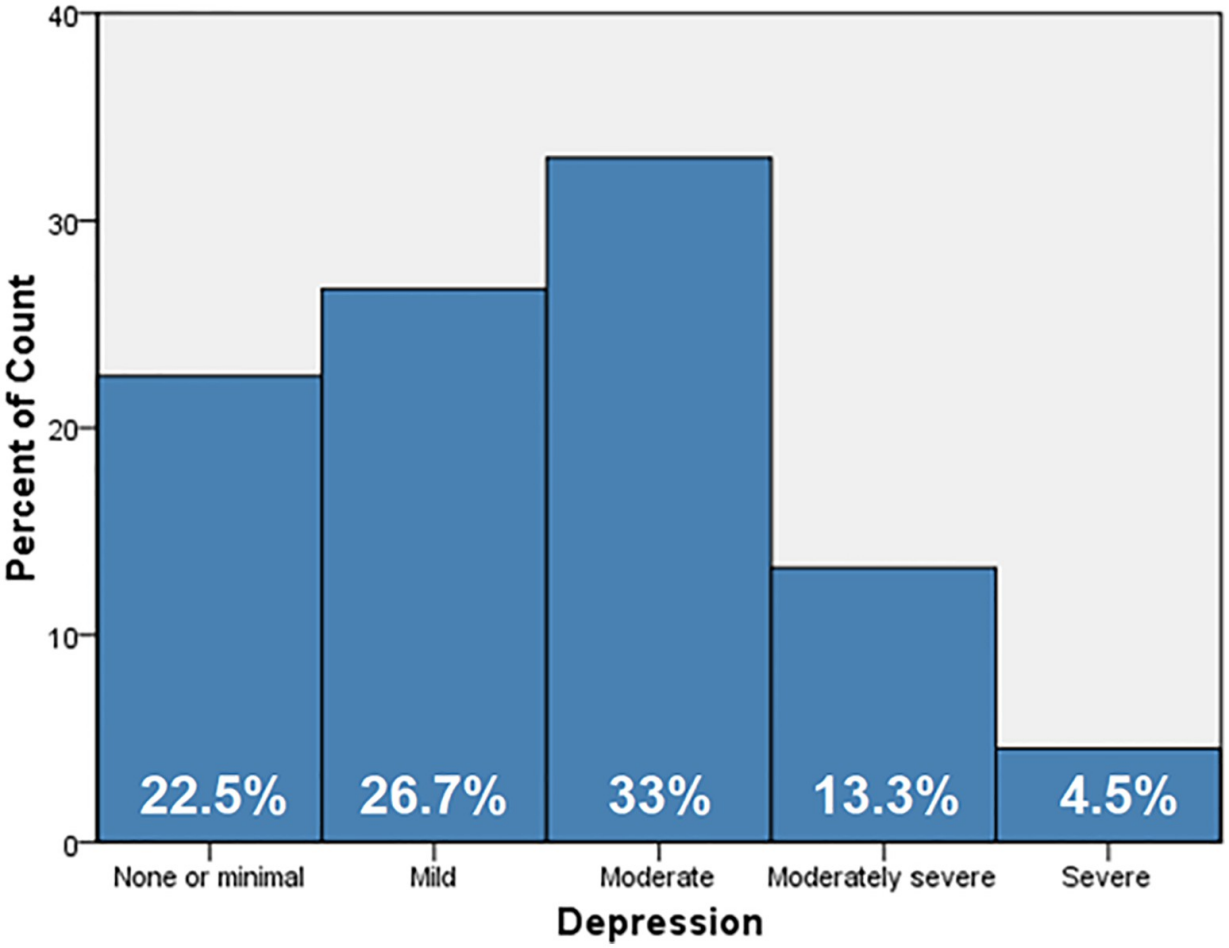
Prevalence of depression among the participants in the study sample.

Table 5 displays the respondent’s answers to the PHQ-9’s nine items. The following was reported as occurring for a few days, more than half the days, or almost every day by these respondents. Feeling down (72.3%); little interest or pleasure in doing things (69%); poor appetite (60.2%); trouble concentrating on things (63.9%); and thoughts that you would be better off dead (33%). According to our data, 22.5% of companions experienced minimum depression, 26.7% had mild depression, 46.3% had moderate and moderately severe depression, and 4.5% had severe depression (Fig 2). To detect the correlation and quantify the relative impact of each independent variable on the total level of depression among the companions, a binary logistic regression analysis was conducted. The logistic regression model was statistically significant, with a p-value<0.001 for X2(9) = 58.17. Tests by Hosmer and Lemeshow: 9.4 (P = 0.307). The Nagelkerke R Square of the model’s explanation of depression -related components was 0.076. The factors that were significantly linked with depression among the companions (P-value<0.05) were gender, occupational status, having children, and maternal status. Females were substantially higher likely to develop depression than males [AOR = 1.322; 95%CI (0.992–1.762)], and companions with hard occupational status were significantly the highest to acquire depression than other companions [AOR = 2.597; 95%CI (1.694–3.981)], in addition the companions who have children were substantially higher likely to develop depression than males [AOR = 1.739; 95%CI (1.299–2.328)], and the divorced companions were significantly the highest to acquire depression than other companions [AOR = 3.929; 95%CI (1.445–10.681)], Table 6.

**Table 5:** Prevalence of depression among the participants in the study sample.

		Frequency	Percentage %
Little interest or pleasure in doing things	Not at all sure	309	31.0%
	Several days	294	29.5%
	Over half the days	230	23.1%
	Nearly every day	163	16.4%
Feeling down, depressed, or hopeless.	Not at all sure	275	27.7%
	Several days	268	27.0%
	Over half the days	242	24.3%
	Nearly every day	209	21.0%
Trouble falling or staying asleep, or sleeping too much	Not at all sure	334	33.6%
	Several days	276	27.7%
	Over half the days	254	25.5%
	Nearly every day	131	13.2%
Feeling tired or having little energy	Not at all sure	226	22.7%
	Several days	326	32.8%
	Over half the days	220	22.1%
	Nearly every day	221	22.2%
	Every day	1	0.1%
Poor appetite or overeating.	Not at all sure	386	38.8%
	Several days	278	27.9%
	Over half the days	197	19.8%
	Nearly every day	135	13.6%
Feeling bad about yourself—or that you are a failure or have let yourself or your family down	Not at all sure	532	53.5%
	Several days	265	26.6%
	Over half the days	121	12.2%
	Nearly every day	76	7.6%
	Every day	1	0.1%
Trouble concentrating on things, such as reading the newspaper or watching television	Not at all sure	360	36.1%
	Several days	318	31.9%
	Over half the days	165	16.6%
	Nearly every day	152	15.3%
	Every day	1	0.1%
Moving or speaking so slowly that other people could have noticed. Or the opposite—being so fidgety or restless that you have been moving around a lot more than usual.	Not at all sure	515	51.7%
	Several days	301	30.2%
	Over half the days	122	12.2%
	Nearly every day	58	5.8%
Thoughts that you would be better off dead, or of hurting yourself in some way	Not at all sure	667	67.0%
	Several days	157	15.8%
	Over half the days	85	8.5%
	Nearly every day	87	8.7%

**Table 6:** Factors associated with depression among the participants in the study sample.

		Depression											
		No Depression		Depression									
		Frequency	Percentage%	Frequency	Percentage%	P-value	COR	Lower	Upper	P-value	AOR	Lower	Upper
Gender	Male	307	30.8%	197	19.8%								
	Female	260	26.1%	232	23.3%	.010	1.391	1.081	1.789	.057	1.322	.992	1.762
Who lives with you?	With Family	537	53.9%	395	39.7%								
	Alone	30	3.0%	34	3.4%	.095	1.541	.927	2.560	.237	1.411	.797	2.497
Smoking	No	325	32.7%	258	25.9%								
	Yes	241	24.2%	171	17.2%	.389	.894	.693	1.154	.577	.921	.689	1.231
Occupational status	Easy	113	11.3%	45	4.5%	.000				.000			
	Moderate	310	31.1%	240	24.1%	.001	1.944	.000	2.043	.000	2.043	1.377	3.030
	Hard	144	14.5%	144	14.5%	.000	2.511	.000	2.597	.000	2.597	1.694	3.981
Do you have children?(	No	429	43.1%	268	26.9%								
	Yes	138	13.9%	161	16.2%	.000	1.868	1.420	2.456	.000	1.739	1.299	2.328
Maternal status	Single	199	20.0%	119	12.0%	.002				.050			
	Married	351	35.3%	280	28.2%	.041	1.334	1.012	1.758	.262	1.187	.880	1.601
	Widower	9	0.9%	11	1.1%	.124	2.044	.823	5.076	.561	1.321	.517	3.379
	Divorced	6	0.6%	19	1.9%	.001	5.296	2.057	13.631	.007	3.929	1.445	10.681

## Discussion

In the present study, we tested for the prevalence, and the associated factors of anxiety and depression among the companions of COVID-19 patients admitted to the ICU. For anxiety, speciality and having a chronic disease were associated with the presence of anxiety in the group subjects. While other sub-groups were not associated with a significant difference in the rates of anxiety. Interestingly, companions living with the family were not associated with a significant difference in the prevalence of anxiety. On the other hand, the prevalence of depression among companions was associated with gender, occupational status, being a parent, and marital status. While smoking and working in the medical field were not associated with a statistically significant change in the prevalence of depression in the study subjects.

Many studies have examined the effect of the COVID-19 pandemic on the mental health of current patients, recovered cases, and family members. Our findings align with studies held in other countries, which indicated a high prevalence of anxiety, depression, and mental health disorders in family members and caregivers of COVID-19 infected subjects [18, 19]. In addition, Orsini et al. [19]. reported an increased incidence of post-traumatic stress, anxiety, and depression in parents whose children were recently diagnosed with COVID-19. They reported an association between economic damage and a higher prevalence of anxiety and depression [19]. These findings don’t support our multivariate logistic regression model, which showed that the wealth index of patients’ companions was not statistical significant predictor of prevalence of anxiety.

Interestingly, wealth index groups were not associated predictors for the presence of depression in our model. These data are understandable, especially in Syria, where the residents have been through 11 years of war and deterioration of the economic situation. In a recent study in Iran conducted during a COVID-19 wave in the country, the prevalence of anxiety was significantly higher among adults aged 30–40 years compared to other age groups, and that is not similar to the pattern observed in our analysis [20].

Our findings showed that females were substantially more likely to develop depression than males because females are known to have a higher prevalence of anxiety and depression disorders because inadequate mental health in women has also been linked to pressure from many social duties and overwork. Compared to males, women are also more likely to experience gender-based discrimination. They could be more likely to have mental health problems as a result. An international study investigating the factors associated with fear and psychological stress during COVID-19 in 17 countries found that females perceive more distress due to changes in employment status, being affected by the change in a financial situation, and the unsure contact with COVID-19 patients [21]. The same study has reported that people living in Syria, Palestine, and Libya, areas of war and conflict, are more associated with moderate to high psychological stress. They also reported that they observed the highest levels of coping stress among Syrian participants compared to the other countries investigated [21]. Similarly, the economic burden has been well studied as a contributing factor to psychological stress. For example, Orsini et al. reported a significant association between anxiety and depressive symptoms and caregivers of children who reported economic damage caused by the COVID-19 pandemic [19]. On the other hand, some studies reported a significant increase in psychiatric distress in young subjects with high monthly incomes. These findings aren’t similar to our findings which showed a statistically significant association between economic level and the presence of both conditions. Many previous studies have reported increased anxiety and depression rates among healthcare workers during a pandemic, especially among nurses [22, 23]. However, a recent meta-analysis concluded that there was no difference between health care workers and the general population in the prevalence of anxiety and depression [24]. This is inconsistent with our results, which showed a strong association between not working in the medical sector and a higher risk of developing depression. It supports the theory that people in the medical industry are more likely to be committed to taking preventative measures that lessen the severity of consequences and are thus more likely to be aware of the possible complications after COVID19.

Anxiety and stress have represented a severe issue during the COVID-19 pandemic. Many interventions were proposed to decrease the burden of psychological stress on both health care workers and the general population. A study conducted on Portuguese nurses has investigated the methods and factors followed by individuals who suffered less anxiety and depression symptoms. Avoiding exposure to news related to the pandemic was significantly associated with lower rates of stress and anxiety. Maintaining social connections and verbalizing emotions were also associated with decreased incidence of anxiety and depression [25]. Another study conducted in Spain among university students has shown that relaxation techniques such as Jacobson’s relaxation techniques and abdominal breathing techniques are associated with a significant decrease in the anxiety scores among these students [26].

## Strengths and limitations

Due to the cross-sectional nature of this research, cause and effect relationships cannot be shown. Unfortunately, we didn’t use a COVID-19-specific tool for evaluating psychological distress. For instance, the Fear of COVID-19 Scale has been determined to be a reliable tool with supporting data [27–30]. Despite the limitations noted, our findings add to the knowledge on the mental anguish experienced by COVID-19 Patient’s Companions, particularly in relation to the frequently occurring mental health issues while providing medical care not only at Aleppo University Hospital but also globally. As the number of COVID-19 patients within the Omicron wave of COVID-19 has decreased, the sample size in this cross-sectional research is adequate for portraying the findings of the population of COVID-19 Patient’s Companions in Syria.

## Conclusion

Our study shows that almost half of the COVID19 patients’ partners report moderate to severe anxiety and sadness. It is crucial to support these groups during the current outbreak (Omicron) since women, those with children, hard workers, and those who are not in the medical profession are more susceptible than others to feel anxious and sadness. Due to the COVID19 pandemic’s current wave and the awful conditions in Syria after an 11-year civil war, Syrian psychological authorities and international organizations should help the people who care for COVID19 patients since many psychological symptoms have been observed among the patients and their companions.

## Supporting information

S1 Dataset (XLSX)
